# Porosity of Rigid Dendrimers in Bulk: Interdendrimer Interactions and Functionality as Key Factors

**DOI:** 10.3390/nano11102600

**Published:** 2021-10-02

**Authors:** Olga Serenko, Kirill Skupov, Artem Bakirov, Nina Kuchkina, Zinaida Shifrina, Aziz Muzafarov

**Affiliations:** 1 A.N. Nesmeyanov Institute of Organoelement Compounds of Russian Academy of Sciences, 28 Vavilova St., GSP-1, V-334, 119991 Moscow, Russia; kskupov@gmail.com (K.S.); kuchkina@ineos.ac.ru (N.K.); z_shifrina@yahoo.com (Z.S.); aziz@ineos.ac.ru (A.M.); 2 N.S. Enikolopov Institute of Synthetic Polymeric Materials, Russian Academy of Sciences, 117393 Moscow, Russia; bakirov.artem@gmail.com

**Keywords:** dendrimer, adsorption, porosity, micropores, π–π-stacking

## Abstract

The porous structure of second- and third-generation polyphenylene-type dendrimers was investigated by adsorption of N_2_, Ar, and CO_2_ gases, scanning electron microscopy and small-angle X-ray spectroscopy. Rigid dendrimers in bulk are microporous and demonstrate a molecular sieve effect. When using CO_2_ as an adsorbate gas, the pore size varies from 0.6 to 0.9 nm. This is most likely due to the distances between dendrimer macromolecules or branches of neighboring dendrimers, whose packing is mostly realized due to intermolecular interactions, in particular, π–π interactions of aromatic fragments. Intermolecular interactions prevent the manifestation of the porosity potential inherent to the molecular 3D structure of third-generation dendrimers, while for the second generation, much higher porosity is observed. The maximum specific surface area for the second-generation dendrimers was 467 m^2^/g when measured by CO_2_ adsorption, indicating that shorter branches of these dendrimers do not provide dense packing. This implies that the possible universal method to create porous materials for all kinds of rigid dendrimers is by a placement of bulky substituents in their outer layer.

## 1. Introduction

Dendrimers are a unique class of monodisperse macromolecules that differ from other polymers by their well-ordered controllable structure and multifunctional periphery. The various structures of dendritic macromolecules as well as the possibility of direct modification of the external dendrimer layer and the internal space of dendrimers determine their numerous possible applications [1,2,3,4,5,6,7,8,9]. The knowledge accumulated to date on dendrimers makes it possible to implement a “from quantity to quality” approach, namely, from expanding the scope of the library of dendritic macromolecules with various chemical structures to the development of new dendrimer-based nanomaterials and discovery of their most promising application areas. Among various applications, the use of dendrimers as porous nanomaterials seems promising, requiring studies of porosity and surface areas of dendrimers and their ensembles.

Rigid-chain dendrimers (for example, polyphenylene types) are thermally stable compounds whose glass transition temperature exceeds the temperature of the onset of the thermal decomposition [10,11]. The synthesis of the phenylene type dendrimers of various chemical structures as well as their molecular characteristics and properties have been extensively reported in several reviews [10,11,12,13]. Taking into consideration the nanometer sizes of these dendrimers and the presence of internal cavities [14,15,16,17,18], one can assume that dendrimer-based nanomaterials would have a highly developed surface. Moreover, because the dendrimer intramolecular-free volume and the type of intermolecular (interdendritic) interactions can be modified, new classes of porous materials with controlled pore sizes can be developed [13,19,20,21,22]. 

The results of the theoretical assessment of the density of polyphenylene dendrimers with “exploded” branching units (~0.03 g/cm^3^ [13,23]) and calculated solvent accessible surface area of a single dendrimer as a structural element of the material [24] reveal the possibility of materials with a high specific surface area based on rigid-chain dendrimers. The specific surface area values obtained in the calculations depend on the generation number and decrease as the latter increases. Nevertheless, the theoretical specific surface area of third-generation “isolated” polyphenylenepyridine dendrimers exceeds 5500 m^2^/g [24]. However, in the study of the specific surface area (*S*) of such dendrimers by the low-temperature adsorption of nitrogen (LTAN), it was found that the *S* value for polyphenylene and polyphenylenepyridine dendrimers of generations one to three did not exceed 100 m^2^/g [24,25], while that for covalent frameworks from phosphorus dendrimers did not exceed 27 m^2^/g [26,27].

According to Lee et al. [28], the cause for the “disappearance” of a porous structure in rigid-chain dendrimers lies in weak van der Waals interactions between dendrimers and nitrogen molecules. As a consequence, the latter are not retained by the surface formed by dendrimers. Another possible explanation of this effect is that micropores formed by dendrimers are not accessible to nitrogen molecules due to dense molecular packing. Rigid-chain aromatic dendrimers are prone to π–π stacking that leads to compacting of the neighboring dendrimers and possible further collapse of the cavities between them [29]. The pores become smaller than it is required for filling with nitrogen molecules or building a monolayer. Thus, the results of the measurements by the low-temperature nitrogen adsorption method may not reflect the real porous structure of these materials. The dominant role of π–π stacking for the formation of ordered structures in case of polyphenylene dendrimers was extensively discussed by Muellen’s group [10,11,12,13,21,22,23,29,30].

CO_2_ can be used as an alternative adsorbate gas for studying the porous structure of nanomaterials. Its molecules are smaller (3.3 Å) and have a larger quadrupole moment (13.4 × 10^−4^ cm^2^) when compared with N_2_ (4.7 × 10^−4^ cm^2^) [31]. This makes CO_2_ sensitive to the presence of polar or charged groups on an adsorbent surface. In fact, the presence of the nitrogen-containing moieties acting as centers of CO_2_ adsorption favors its adsorption by the adsorbent [31,32,33,34,35,36,37,38,39]. Unlike low-temperature N_2_ adsorption (77 K), CO_2_ adsorption is usually measured at 273 K, which leads to a higher diffusion rate of this gas and provides a better penetration into micropores [31]. According to Lee et al., a replacement of N_2_ as the adsorbate gas with CO_2_ leads to a sharp increase in the amount of gas adsorbed by dendrimers containing rigid NH-triazine fragments [28].

However, to date the important issue of whether rigid-chain dendrimers can form highly porous materials due to their molecular architecture and whether their porous structure can be controlled remains unresolved. In this paper we are addressing this issue, carrying out the study of the adsorption properties of porous structures based on rigid-chain dendrimers of various chemical compositions and generation numbers using N_2_, Ar, and CO_2_ as the adsorbates. We demonstrated that interdendrimer interactions, which provide dendrimer packing is a main factor, leading to the absence or presence of porosity in dendrimers depending on the generation. We believe that the synthesis of rigid-chain dendrimers with bulky terminal groups could be a universal path for all rigid dendrimers to develop highly porous materials.

## 2. Materials and Methods

### 2.1. Dendrimers

**Figure 1:**
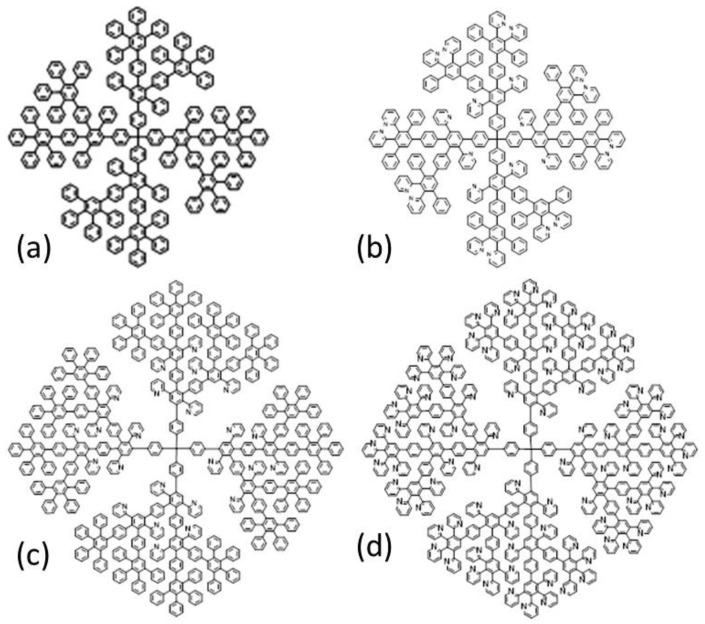
Structural formulas of dendrimers: G2-PhPh (**a**), G2-PyPy (**b**), G3-PyPyPh (**c**) and G3-PyPyPy (**d**).

We used second- and third-generation dendrimers of the polyphenylene type, the synthesis of which is described elsewhere [14,15,23]. Their structural formulas are shown in Figure 1. Dendrimers differ by the presence or absence of pyridine fragments and by their distribution within the dendrimer molecules. They are designated as G2-PhPh, G2-PyPy, G3-PyPyPh, G3-PyPyPy. The labels indicate the chemical structure of the dendrimer layers, phenylene (Ph) or pyridyl (Py), and their sequence in the dendrimer framework.

Second- and third-generation dendrimers have hydrodynamic radii 1.5 nm [23] and 3.6 nm [40] for G2-PhPh and G3-PyPyPh, respectively. The G3-PyPyPy diameter determined by the Atomic Force Microscopy (AFM) equals 5.5 nm [15].

### 2.2. Characterization

Skeletal density of dendrimers (*ρ_s_*) was determined using an AccuPyc 1340 automatic helium pycnometer (Micromeritics Instrument Corporation, 4356 Communications Drive, Norcross, GA 30093, USA).

The bulk density of dendrimer G3-PyPyPy (*ρ* = weight/volume) was determined using 1 mL pycnometer.

The morphology of the dendrimer powders was investigated by performing scanning microscopy (SEM) with a JEOL JSM-6510LV microscope (JEOL, Tokyo, Japan). 

N_2_ and CO_2_ adsorption–desorption isotherms were obtained at 77 and 273 K, respectively, with the Surface Area and Pore Size Analyzer System 3P Micro 200 (3P Instruments GmbH & Co. KG, Odelzhausen, Germany). Before the measurements, the samples were degassed at 423 K for 16 h until the residual pressure reached 5 Pa. For nitrogen adsorption, the calculations were performed by the BET method. For higher precision, Rouquerol consistency criteria were applied. The BET equation was applied to a part of the isotherm when *V*(1 − *p*/*p_0_*) is increasing with *p*/*p*_0_ and precisely limited by *p*/*p_0_* value at the maximum of the Rouquerol plot V(1 − *p*/*p_0_*) vs. *p*/*p_0_* (*V* is the volume of adsorbed nitrogen, *p_0_* is thesaturation pressure) [40]. For CO_2_ adsorption, the calculations were performed according to Dubinin-Radushkevich (DR), Non-Linear Density Functional Theory (NLDFT) and Grand Canonical Monte Carlo (GCMC) methods.

The measurement of the specific surface area when using argon as an adsorbate was carried out on a Tsvet 211 sorption meter (St. Petersburg, Russia) according to the described technique [41] (see the Supplementary Materials). Specific surface areas were calculated from the desorption data using the BET method.

High resolution small-angle diffraction patterns were recorded using S3-Micropix camera, manufactured by Hecus X-Ray Systems (Graz, Austria) (CuKα radiation—*λ* = 1.542 Å). Two detectors were employed: a two-dimensional Pilatus 100 K detector and a linear gas position sensitive detector PSD 50M operating at a pressure of 8 bar Ar/Me. For shaping of the X-ray beam, the Fox 3D vacuum optics were used. The slits in the Kratky collimator were set to 0.1 and 0.2 mm, respectively. The angular scale was between 0.003 Å^−1^ and 1.9 Å^−1^. To calibrate small- and wide-angle diffractograms, silver behenate and lupolen (LDPE) calibrants were used as references. To eliminate the influence of air, the X-ray optics system and the camera were vacuumed to pressure (2 ÷ 3) × 10^−2^ mm Hg. The exposition time was varied from 600 to 3000 s.

## 3. Results

**Figure 2:**
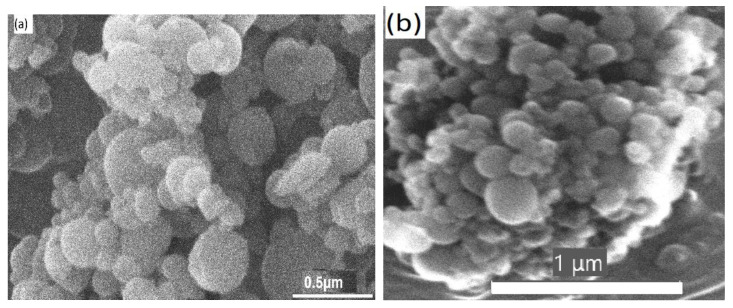
Surface morphology of dendrimer powders: G2-PhPh (**a**) and G3-PyPyPh (**b**).

Rigid-chain dendrimers in bulk are amorphous powders and have different skeletal densities (see Table S1). Their SEM micrographs are shown in Figure 2 and Figure S1. The particles observed are aggregates of smaller spherical submicron particles, which, in turn, should be formed by packed dendrimers. Analysis of the micrographs shows that the surface of submicron spherical particles has no defects. The diameters of submicron particles vary from 100 to 450 nm for G2-PhPh, from 50 to 200 nm for G2-PhPy, and from 100 to 250 nm for G3-PyPyPh, indicating the formation of loose dendrimer aggregates. Conversely, dendrimer G3-PyPyPy forms dense, shapeless aggregates, consisting of spherical particles with diameters 100–150 nm [24]. The largest variation in sizes of spherical particles is observed for G2-PhPh.

Table 1 displays the surface characteristics of dendrimers calculated from the isotherms of low-temperature nitrogen adsorption that are presented in Figure S2. The data show that the largest S values are 95 and 98 m^2^/g for G2-PhPy and G3-PyPyPh, respectively, while the smallest values are 33 and 17 m^2^/g for G2-PhPh and G3-PyPyPy, respectively. Analysis of experimental data by the t-plot method has shown no micropores in the dendrimer powders if N_2_ is used as the adsorbate.

The replacement of N_2_ with Ar, whose molecules are smaller than those of N_2_, namely, 3.4 Å for Ar and 3.64 Å for N_2_, did not lead to an increase in the porosity value. The BET S values of G2-PyPy and G3-PyPyPy were 71 and 47 m^2^/g, respectively.

These low porosity values and the lack of micropores for these samples were surprising. This stimulated us to employ SAXS for porosity measurements, because according to literature [42], it should provide more accurate *S* values. The G3-PyPyPy sample was chosen for this experiment. 

The procedure for calculating the total specific internal surface from SAXS data according to Porod’s approximation is described in Ref. [43].

The specific surface area value (*S*/*m*, m^2^/g) was calculated using the following Porod equation:

$$\frac{S}{m}=\frac{\pi k\varphi \left(1-\varphi \right)}{\rho_{s}Q}$$

where *ρ_c_* is the skeletal density of the sample, *k* is the Porod constant, *ϕ* is the pore volume fraction in the sample and *Q* is the invariant. 

The volume fraction of pores (*φ*) in the sample required for calculating of *S* was determined as $\varphi =1-\frac{\rho }{\rho s}$ [44]. Here, *ρ* is the powder bulk density equal to 0.536 g/cm^3^; *ρ_s_* is the skeletal density equal to 1.286 g/cm^3^ as measured by the helium pycnometry method. Calculated *k*/*Q* is 0.0088 (Figure S3). The newly obtained *S* value for this dendrimer is 52 m^2^/g, which matches the data obtained by the adsorption measurements.

**Figure 3:**
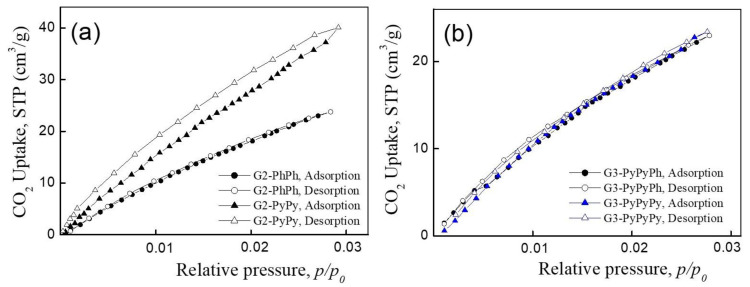
CO_2_ adsorption—desorption isotherms for dendrimers of second (**a**) and third (**b**) generations.

To understand this phenomenon, we explored a different adsorbate gas (CO_2_) whose diffusion rate at 273 K is higher than that of N_2_ at 77 K [31]. This should facilitate the penetration of CO_2_ into micropores, allowing for higher specific surface areas, if the cause of low *S* values lies in limited diffusion. Figure 3 shows the adsorption–desorption isotherms of CO_2_ on dendrimer powders at *p*/*p_0_* ≤ 0.03. The adsorption–desorption curves of G2-PhPy lie higher than those of G2-PhPh. For G2-PhPy, the desorption branch of the isotherm lies above the sorption branch, forming the hysteresis loop and indicating the delayed desorption due to higher CO_2_ uptake. For G2-PhPh, the sorption and desorption curves coincide. Third-generation dendrimers, G3-PyPyPh and G3-PyPyPy, showed no hysteresis loops either.

To calculate the parameters of the porous structure of dendrimers from CO_2_ adsorption isotherms, we used a method based on the micropore volume filling theory by Dubinin-Radushkevich, as well as the Non-Linear Density Functional Theory (NLDFT) and Grand Canonical Monte Carlo (GCMC) methods. The choice of these methods instead of the BET was based on the fact that the adsorption and micropore volume filling processes are indistinguishable and the formation of a monolayer or polymolecular (multilayer) adsorption in the *p*/*p_0_* range from 0 to 0.03 is unlikely [45].

Table 1 shows the characteristics of dendrimer powders calculated from the CO_2_ adsorption data by the above methods. The newly obtained specific surface areas differ significantly from the LTAN data. For example, the *S* of G2-PhPy which was 95 m^2^/g with nitrogen as the adsorbate, increased to 467 m^2^/g in case of CO_2_. Similarly, the specific surface area of G3-PyPyPy increased from 17 m^2^/g to 281 m^2^/g.

It is noteworthy that the pore sizes in dendrimers are 0.6–0.9 nm. They belong to micropores and are smaller than the dendrimer molecules themselves. Some authors attribute pores that are smaller than 1 nm to ultra- and supermicropores [46]. These pores most likely represent a distance between the dendrimers or between the branches of the adjacent dendrimers that is determined by the mechanism of their assembly, in particular, by the π–π stacking interactions.

A comparison of the parameters of porous structures of two second generation dendrimers with and without pyridyl units using the CO_2_ adsorption, allows us to propose that the larger specific surface area of G2-PhPy than that of G2-PhPh is associated with the presence of pyridine groups, since the nitrogen-containing moieties in an adsorbent increase the adsorption of CO_2_, whose molecules possess a quadrupole moment [31,32,33,34,35,36,37,38,39].

The question arises is the “pyridine group factor” determines the CO_2_ adsorption for higher (third) generation dendrimers? To our surprise, this was not the case, although enhanced CO_2_ adsorption on pyridine groups should be the same. Indeed, the S values of G3-PyPyPy and G3-PyPyPh (Table 1) differ by less than 10% (according to DFT data) and the other parameters are also quite similar. A difference in S or V calculated by the GCMC method does not exceed 10% or 15%, respectively. Thus, there is no effect on porosity from the increased generation number, the chemical structure of dendrimers, including additional pyridine groups in the outer layer, and the morphology of the aggregates observed by SEM. This phenomenon can be assigned to denser packing of the third-generation dendrimers in bulk due to longer branches. 

A combination of three factors: (i) the dependence of the porosity parameters on the type of adsorbate (i.e., when the adsorbate is changed, the *S* values of the adsorbent may change significantly), (ii) the detection of micropores in all the dendrimers with the use of CO_2_ adsorbate, and (iii) the weak effect of the nitrogen-containing moieties on the porosity parameters reveals that rigid-chain polyphenylene-type dendrimers act as molecular sieves where the micropore widths are close to the dimension of the adsorbate. A similar effect was observed previously in studies of coals [34,47,48], fullerenes [49], COFs [50], and MOFs [51].

While the G2-PhPh, G3-PyPyPy and G3-PyPyPh dendrimers have similar porosity characteristics, G2-PhPy differs from them and has the highest *S* and *V* values. The question arises why such a difference is observed? We propose that the microporous structure of powders is “localized” in submicron particles formed by the dendrimers (Figure 2). When micropores are smaller than 1 nm, the accessibility of the pores for an adsorbate gas is a crucial factor. Thus, the diffusion rate of the adsorbate, the ratio of the pore diameter to the diameter of adsorbate molecules, as well as a “filling rate” of pores are important to determine the S. According to Ismail et al. [42], the complete filling of pores, the size of which is approximately three diameters of the adsorbate molecule, is unlikely. In the case of dendrimers studied here, this ratio varies 1.6 to 2.6, which is less than the value estimated by Ismail et al. [49]. Therefore, the *S* and *V* values found may not reflect the real porosity of rigid-chain dendrimers and may be bigger if gases whose molecules are smaller than CO_2_ are used, such as H_2_ (molecule size of 2.8 A), Ne (2.4 A), or He (2.0 or 1.95 A). Certainly, this assumption requires an experiential verification since the adsorption of each gas mentioned above and its diffusion into the micropores have some specific features [48,52].

## 4. Discussion

Based on the hypothesis that the porous structure of dendrimer powders is “embedded” in denser submicron globules of powder particles, the protocol of their formation may change their morphology, and hence, the porosity. We believe the assembly of particles from individual dendrimers to give submicron globules occurs either by successive “addition” of dendrimers to an assembly of these particles (particle-cluster type aggregation) or by integration of clusters consisting of dendrimers (cluster-cluster type aggregation). As was demonstrated earlier, G2-PhPy dendrimers form stable clusters in solution with sizes exceeding the typical sizes of single macromolecules [40]. In this case, it is highly likely that the growth of aggregates from G2-PhPy would occur by integration of clusters and small aggregates. This could lead to the formation of submicron particles with a larger porosity or particles with pores, whose channel geometry does not prevent their complete filling with an adsorbate gas. This increases the amount of the adsorbed gas and the estimated specific surface area. This assumption is based on the data reported for silica gel particles. It was demonstrated that the growth of silica gel particles by addition of an “elementary structural unit” to a preformed particle results in dense globules with low porosity, while linking of the preformed aggregates gives loose, highly porous particles [53,54,55,56].

Based on the data on coagulation of nanosized particles [55,56,57], we hypothesize that by changing the solvent (including supercritical fluids) and the type of the “anti-solvent” acting as a coagulant, the solvent concentration, conditions of drying the resulting powders, etc., it should be possible to control the material porous structure for the porosity increase. However, we believe that these actions will not lead to the implementation of the porosity potential inherent to the molecular 3D-structure of dendrimers (see the Introduction) because they do not provide a control of the intermolecular interactions, in particular, intermolecular π–π stacking which favors the ordering of dendrimers into a dense molecular architecture. To prevent the undesirable intermolecular interactions, an attachment of bulky terminal groups to rigid-chain dendrimers might be explored.

## 5. Conclusions

Rigid-chain dendrimers of polyphenylene type are characterized by microporosity and demonstrate the molecular sieve effect. The use of CO_2_ as an adsorbate gas that can penetrate into small micropores more effectively at 273 K than N_2_ and Ar at 77 K allowed us to measure the size of dendrimer micropores in the bulk, which vary in the range from 0.6 to 0.9 nm. 

The porous structure of dendrimers in bulk is determined by the morphology of submicron globules formed by individual dendrimers. We believe, the conditions of their assembly may affect the density of submicron globules, and hence, the porosity of dendrimer assemblies.

The implementation of controlled interdendritic interactions can be considered as the promising synthetic strategy for the development of new highly porous materials based on dendrimers. The knowledge of the dendrimer chemistry including the crucial role of the external dendrimer layer as well as its contribution to dendrimer properties gives one an opportunity to solve this problem. It is quite likely bulky substituents of the external dendrimer layer will prevent the collapse of the free volume of 3D-molecular structures and could be a promising avenue for future design of porous materials based on rigid dendrimers.

## Figures and Tables

**Table 1:** Adsorption properties of dendrimers *.

Dendrimer	N_2_ Adsorbate		CO_2_ Adsorbate							
			Method							
	Pore Volume, cm^3^/g	*S_BET_*, m^2^/g	Dubinin-Radushkevich (DR)		(Non-Linear) Density Functional Theory (DFT)			Grand Canonical Monte Carlo (GCMC)		
			Micro-pore Volume, cm^3^/g	Average Micro- Pore Size, nm	Micro- Pore Volume, cm^3^/g	Average Micro- Pore Size, nm	*S_DFT_*, m^2^/g	Micro- Pore Volume, cm^3^/g	Average Micro- Pore Size, nm	*S_GCNC_*, m^2^/g
G2-PhPh	0.128	33	0.150	1.2	0.095	0.6	257	0.120	0.9	292
G2-PyPy	0.909	95	0.239	1.3	0.158	0.6	**420**	0.198	0.8	**467**
G3-PyPyPh	0.302	98	0.148	1.2	0.082	0.6	236	0.099	0.6	254
G3-PyPyPy	0.141	17	0.144	1.2	0.068	0.5	215	0.114	0.6	281

* Calculations were based on adsorption data.

## Supplementary Materials

The following data are available online at https://www.mdpi.com/article/10.3390/nano11102600/s1, Figure S1: Surface morphology of dendrimer powders: G2-PyPy (a) and G3-PyPyPy (b); Figure S2: N_2_ adsorption–desorption isotherms on dendrimers of second (a) and third (b) generations; Figure S3: SAXS curve for G3-PyPyPy in Porod coordinates. The Porod constant is defined as the point of intersection of the ordinate axis with the linear approximation of the wide-angle region of the SAXS curve; Table S1. Skeletal density of dendrimers (*ρ_s_*).
