# Supplementary material for: Porosity of Rigid Dendrimers in Bulk: Interdendrimer Interactions and Functionality as Key Factors
Source: Nanomaterials (Basel). 2021 Oct 2;11(10):2600. doi: 10.3390/nano11102600 (PMC8537161; doi:10.3390/nano11102600)
Supplement: Supplementary file 1 [file nanomaterials-11-02600-s001.zip › nanomaterials-1399193-supplementary.pdf]

nanomaterials

Supplementary Material

Porosity of Rigid Dendrimers in Bulk: Interdendrimer Interactions and Functionality as Key Factors

Olga Serenko [1,*], Kirill Skupov [1], Artem Bakirov [2], Nina Kuchkina [1], Zinaida Shifrina [1] and Aziz Muzafarov [1]

[1] A.N. Nesmeyanov Institute of Organoelement Compounds of Russian Academy of Sciences, 28 Vavilova St., GSP-1, V-334, 119991 Moscow, Russia; kskupov@gmail.com (K.S.); kuchkina@ineos.ac.ru (N.K.); z_shifrina@yahoo.com (Z.S.); aziz@ineos.ac.ru (A.M.)
[2] N.S. Enikolopov Institute of Synthetic Polymeric Materials, Russian Academy of Sciences, 117393 Moscow, Russia; bakirov.artem@gmail.com
* Correspondence: oserenko@yandex.ru

Citation: Serenko, O.; Skupov, K.; Bakirov, A.; Kuchkina, N.; Shifrina, Z.; Muzafarov, A. Porosity of Rigid Dendrimers in Bulk: Interdendrimer Interactions and Functionality as Key Factors. *Nanomaterials* **2021**, *11*, 2600. https://doi.org/10.3390/nano11102600

Academic Editor: Jørn B. Christensen

Received: 13 September 2021
Accepted: 30 September 2021
Published: 2 October 2021

Publisher's Note: MDPI stays neutral with regard to jurisdictional claims in published maps and institutional affiliations.

Copyright: © 2021 by the authors. Licensee MDPI, Basel, Switzerland. This article is an open access article distributed under the terms and conditions of the Creative Commons Attribution (CC BY) license (https://creativecommons.org/licenses/by/4.0/).

When using argon as an adsorbate gas the value of the surface area was calculated according to the BET theory using the Temkin equation for the capacity of monolayer:

$$V_m = V(1 - {}^{p}/_{p_s})(1 + \frac{{}^{p_s}/_{p} - 1}{C}) \quad (1)$$

where V_m is the capacity of monolayer, cm³ /g; V is the value of adsorption corresponding to the given relative pressure p/p_s, cm³/g; p is the equilibrium pressure of argon, mm Hg; p_s is the pressure of argon saturated vapor at T = –196°C and p_s = 240 mm Hg; and C is the *BET* constant depending on the energy of adsorption and temperature (in calculations the value of C was taken to be 60) [41]. The apparent BET surface area was calculated according to the equation:

$$S = 4.17V_m \quad (2)$$

where 4.17 is the area occupied by 1 mL of argon poured as a monomolecular layer.

Table S1. Skeletal density of dendrimers (ρ_s).

Dendrimer	ρ_s, g/cm³
G2-PhPh	0.879
G2-PyPy	0.942
G3-PyPyPh	1.123
G3-PyPyPy	1.286

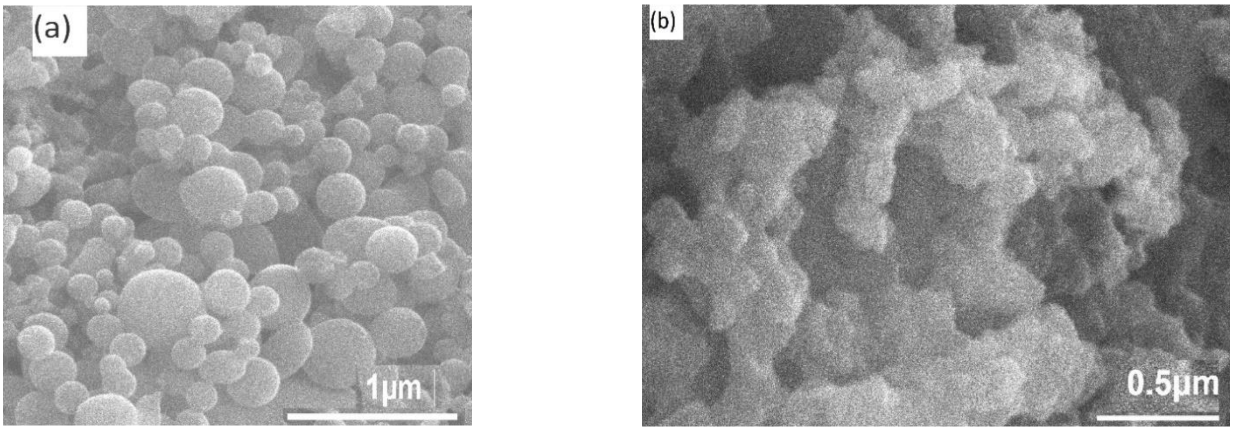

Figure S1. Surface morphology of dendrimer powders: G2-PyPy (**a**), and G3-PyPyPy (**b**).

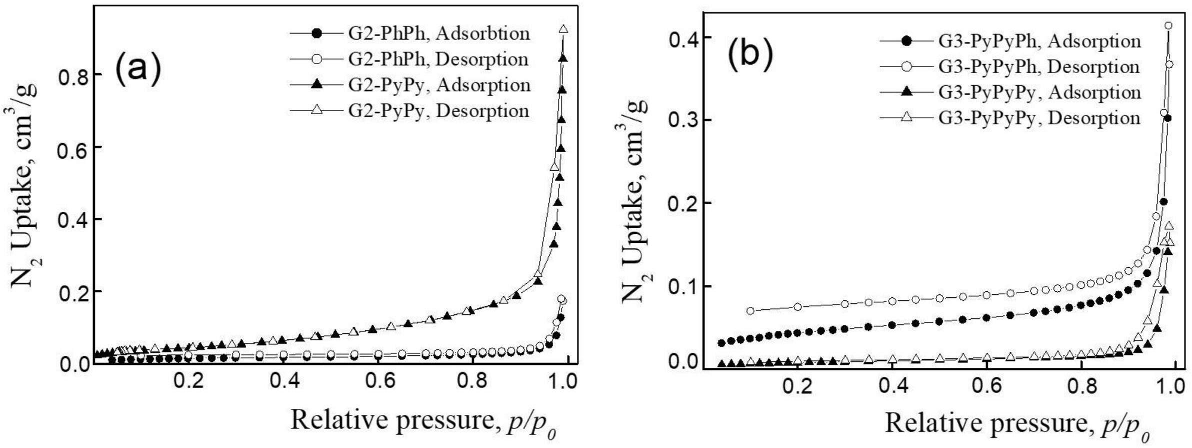

Figure S2. N_2 adsorption–desorption isotherms on dendrimers of second (**a**), and third (**b**) generations.

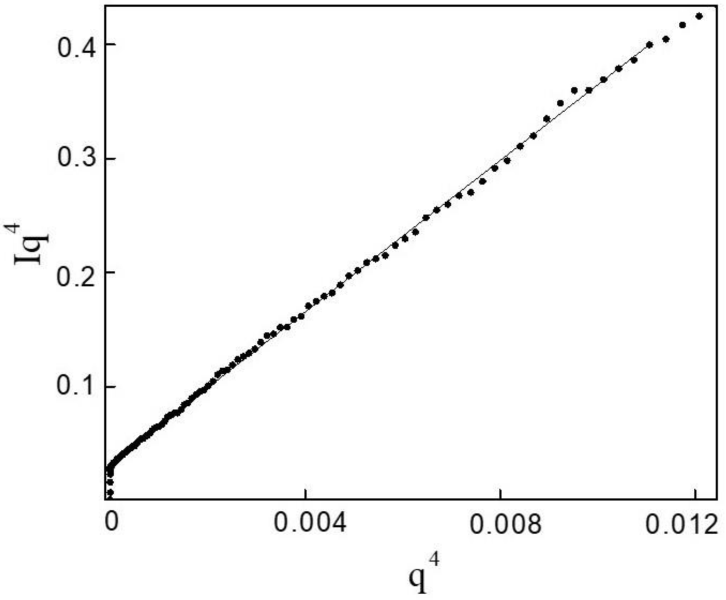

Figure S3. SAXS curve for G3-PyPyPy in Porod coordinates. The Porod constant is defined as the point of intersection of the ordinate axis with the linear approximation of the wide-angle region of the SAXS curve.
